# Late follow-up of neo-aortic dimensions and coronary arteries in adult patients after the arterial switch operation^☆^

**DOI:** 10.1016/j.ijcchd.2023.100481

**Published:** 2023-10-14

**Authors:** Diederick B.H. Verheijen, Leo J. Engele, Anastasia D. Egorova, J. Lauran Stöger, Bart J.A. Mertens, Roel L.F. van der Palen, Dave R. Koolbergen, Mark G. Hazekamp, J. Wouter Jukema, Hubert W. Vliegen, Berto J. Bouma, Monique R.M. Jongbloed, Philippine Kiès

**Affiliations:** a CAHAL, Center for Congenital Heart Disease Amsterdam-Leiden, Location Leiden University Medical Center, the Netherlands; b Department of Cardiology, Leiden University Medical Center, Leiden, the Netherlands; c Department of Cardiology, Amsterdam University Medical Center, Amsterdam, the Netherlands; d Department of Radiology, Leiden University Medical Center, Leiden, the Netherlands; e Department of Medical Statistics, Leiden University Medical Center, Leiden, the Netherlands; f Department of Pediatrics, Division of Pediatric Cardiology, Leiden University Medical Center, Leiden, the Netherlands; g Department of Cardiothoracic Surgery, Leiden University Medical Center, Leiden, the Netherlands; h Netherlands Heart Institute, Utrecht, the Netherlands; i Department of Anatomy & Embryology, Leiden University Medical Center, Leiden, the Netherlands

**Keywords:** Transposition of the great arteries, Arterial switch operation, Neo-aortic dilatation, Acute take-off angle, Computed tomography angiography

## Abstract

**Background:**

After the arterial switch operation (ASO) for transposition of the great arteries (TGA), neo-aortic dilatation and coronary arterial anomalies, especially an interarterial course and acute coronary artery take-off angle, are commonly found. Long-term follow-up data after ASO is scarce. Aim of this study was to determine the prevalence of neo-aortic dilatation and coronary abnormalities, with special emphasis on acute coronary take-off angle, in adult TGA-ASO patients.

**Methods:**

In this retrospective cohort study, all adult TGA-ASO patients with ≥1 CT-angiography (CTA) at the age of ≥16 years were included.

**Results:**

Eighty-one patients, 69 % male and median age 21.0 [18.5–22.8] years, were included. At baseline, maximum neo-aortic diameter was 39.2 ± 5.3 mm; 35 (43 %) patients had neo-aortic dilatation (neo-aortic diameter of >40 mm), 22 (27 %) patients had an acute coronary take-off angle (<30°), and 5 (6 %) patients had an interarterial course of the RCA (2 %) or LCA (4 %). Neo-aortic or coronary artery re-intervention occurred in 10 (12 %) patients. All 10 patients had neo-aortic dilatation or coronary take-off angle of <30° on baseline CTA.

**Conclusion:**

This study reports a prevalence of 43 % of neo-aortic dilatation, 6 % of interarterial coronary course and 27 % for acute coronary take-off angle (<30°) at a median term of 21.0 years post ASO. All patients with a neo-aortic re-intervention or coronary artery re-intervention during follow-up had a maximum neo-aortic diameter of >40 mm or a coronary take-off angle of <30° at baseline CTA. This hypothesis generating study suggests that an active surveillance in patients with neo-aortic dilation and/or an acute angulation of < 30° post ASO might be considered and requires prospective evaluation.

## Graphical abstract

**Figure:**
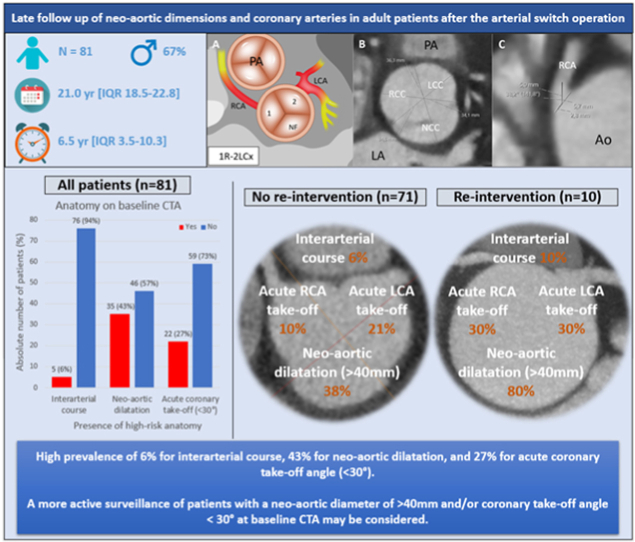

## Abbreviations

ASO: Arterial switch operation
BMI: Body mass index
BSA: Body surface area
CABG: Coronary artery bypass graft
CAG: Coronary angiography
Cx: Circumflex artery
CTA: Computed tomography angiography
DORV: Double outlet right ventricle
DORV-TBA: DORV of the Taussig-Bing type
HU: Hounsfield units
ICC: Interclass correlation coefficient
ICD: Implantable cardioverter-defibrillator
IVS: Intact ventricular septum
LAD: Left anterior descending artery
LCA: Left coronary artery
PCI: Percutaneous coronary intervention
RCA: Right coronary artery
SPECT: Single-photon emission computerized tomography
TGA: Transposition of the great arteries
VSD: Ventricular septum defect

## 1 Introduction

Transposition of the great arteries (TGA) is a relatively common cyanotic heart defect and accounts for 5–7% of all patients with congenital heart disease [1]. The arterial switch operation (ASO) is currently the preferred surgical technique in TGA patients with suitable anatomy and has been proven superior to the atrial switch operation in most cases [2,3]. During the ASO, transfer of the coronary arteries to the neo-aorta is a critical step and problems with translocation of the coronary arteries are associated with early mortality and morbidity [4]. Long-term patency of the re-implanted coronary arteries remains a concern and optimal follow-up strategy during adulthood for evaluation of the patency of the reimplanted coronary arteries is not determined [5].

Previous studies reported that the presence of high-risk anatomical features of the coronary arteries, including an interatrial course and acute take-off angle, is common in TGA-ASO patients [6,7]. However, the clinical consequences of these high-risk features in TGA patients are unknown and follow-up duration in this young adult ASO population is still limited [8].

Furthermore, dilatation of the neo-aortic root due to the systemic pressure exerted on the native pulmonary root tissue is a well-known long-term complication after ASO [7]. Dilatation of the neo-aortic root has been reported as a risk factor for the development of neo-aortic valve regurgitation during long-term follow-up [9]. However, whether the ongoing neo-aortic dilatation and valve regurgitation will result in increased number of neo-aortic root or valve intervention during adulthood is largely unknown.

The primary aim of the current study is to determine the prevalence of neo-aortic dilatation, interarterial coronary course and acute coronary take-off angle in adult TGA-ASO patients, taking into account whether patients had a re-intervention during follow-up.

## 2 Methods

This retrospective cohort study was conducted at the tertiary referral Center for Congenital Heart Disease Amsterdam-Leiden (CAHAL), location Leiden University Medical Center. All consecutive adolescent and adult TGA-ASO patients were eligible for inclusion if at least 1 computed tomography angiography (CTA) of the coronary arteries was performed ≥16 years of age. Patients were excluded if they had a bicuspid neo-aortic valve or if they had undergone a coronary artery or neo-aortic root intervention prior to the first CTA. Demographic and clinical data was collected from the electronic health record systems.

Baseline data was retrieved at the time of the first CTA. Body surface area (BSA) was calculated using the Dubois and Dubois formula [10]. CTA measurements were performed independently by two experienced observers (DBHV and LJE). All cardiovascular events, including cardiovascular interventions, were evaluated until the latest follow-up (closing date of follow-up October 15, 2022).

### 2.1 Computed tomography angiography

**Fig. 1:**
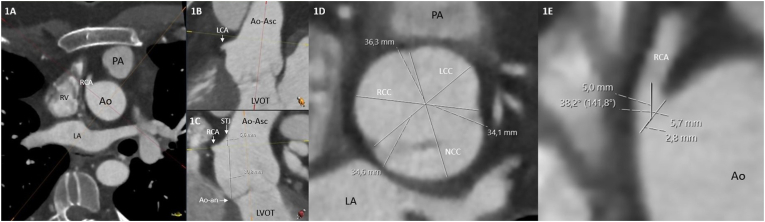
CTA analysis approach. A–C: 3-dimensional multiplanar reconstruction with double oblique planes perpendicular to the neo-aortic annulus. C: 3-dimensional multiplanar reconstruction plane to additionally measure the distance from the coronary ostium to the neo-aortic annulus. D: Cusp-to-commissure diameter measurements of the neo-aortic root. E: Measurement of the coronary take-off angle. Abbreviations: Ao, aorta; Ao-an, neo-aortic annulus; Ao-Asc, ascending aorta; CTA, computed tomography angiography; LA, left atrium; LCA, left coronary artery; LCC, left coronary cusp; LVOT, left ventricular outflow tract; NCC, non-coronary cusp; PA, pulmonary artery; RCA, right coronary artery; RCC, right coronary cusp; RV, right ventricle; STJ, sinotubular junction.

Prospective ECG-triggered CTA with a slice thickness of 0.5–0.6 mm were used for analysis. Patient preparation was done according to the Society of Cardiovascular Computed Tomography guidelines [11]. The images were analyzed in the diastolic phase at 70–80 % of the R–R interval, at the width of 1200 Hounsfield units (HU) and level of 300 HU. The methodology of the CTA measurements is illustrated in Fig. 1. Coronary anatomy (Fig. 1A) was classified according to the Leiden Convention coronary coding system [12]. The postoperative 1R-2LCx variant in which the right coronary artery (RCA) originates from ‘sinus 1’ as defined by Gittenberger-de Groot et al. and the left coronary artery (LCA) from ‘sinus 2’ post ASO, was classified as ‘common coronary anatomy’ (pre-operative coronary anatomy was in these cases 1LCx-2R) and all other variants were classified as ‘variant coronary anatomy’ [7,13]. In the same view, presence of an interarterial course was assessed. An interarterial course was defined as a coronary take-off or course in between the aorta and pulmonary trunk. To assess the coronary take-off height, a 3-dimensional multiplanar reconstruction with double oblique planes was created parallel to the centerline of the aorta and perpendicular to the neo-aortic valve leaflets (Fig. 1A–C). In this view the distance from the coronary ostium to the neo-aortic annulus (Fig. 1B) was measured. Subsequently, the neo-aortic dimensions were measured at the level of the neo-aortic annulus, neo-aortic sinus, RCA ostium, LCA ostium, sinotubular junction and ascending aorta. At each level, 3 cusp-to-commissure measurements (commissure to right coronary cusp, commissure to left coronary cusp and commissure to non-coronary cusp) or wall-to-wall measurements were obtained (Fig. 1D) [14]. Absolute and BSA indexed aortic diameters for each level and the maximum neo-aortic diameters are presented. Aortic dilatation was defined as an absolute diameter of >40 mm [15,16]. For measurements of the coronary take-off angle, a 3-dimensional multiplanar reconstruction with double oblique planes parallel to the centerline of the proximal coronary artery was created. Subsequently, the take-off angle was measured between the coronary base tangent to the aorta and an imaginary straight line of 5 mm from the coronary ostium to a point along the centerline of the coronary artery, by taking into account the 3-dimensional course and angle of the coronary artery [17,18](Fig. 1E). In the current literature, two different cut-off values for an acute take-off angle are defined, therefore, analyses were performed with both the cut-off values of <30° and <45° [7,17].

### 2.2 Statistical analysis

Statistical analysis was performed using SPSS (version 25; SPSS Inc, Chicago, USA). Evaluation of normal distribution of continuous data was assessed using the Shapiro–Wilk test. Normally distributed continuous data were presented as mean ± standard deviation (SD) and non-normally distributed continuous data were presented as median and the first to third interquartile range [IQR1-IQR3]. Categorical data were presented as absolute numbers and percentages (%). Differences between patients with ‘no re-intervention’ and with a ‘re-intervention’ were assessed with the Mann-Whitney *U* test and Fisher's Exact test. Differences between the baseline and follow-up CTA were assessed with the Wilcoxon Signed Rank test and Fisher's Exact test. Pearson's correlation was used to assess the relation between the neo-aortic dimensions and the coronary take-off angle. A p-value of <0.05 was considered statistically significant.

An interobserver agreement was calculated for coronary take-off angle measurements and neo-aortic dimensions. Individual and independent measurements between DBHV and LJE were evaluated by calculating the intraclass correlation coefficient (ICC) in a two-way mixed model with a confidence interval of 95 %. ICC values of <0.5, 0.5–0.75, 0.75–0.9 and > 0.9 indicated poor, moderate, good and excellent reliability, respectively [19].

## 3 Ethics statement

All tests and procedures performed involving human participants were in accordance with the ethical standards of the institutional and national research committee and with the 2013 Helsinki declaration or comparable ethical standards. Appropriate local scientific board approval (Department of Cardiology, Leiden University Medical Center, the Netherlands) was obtained, and the need for written informed consent was waived by the institutional medical ethical board, The Medical Ethics Committee Leiden, The Hague, Delft (protocol reference number G21.201, 14-12-2021).

## 4 Results

### 4.1 Patients characteristics

**Fig. 2:**
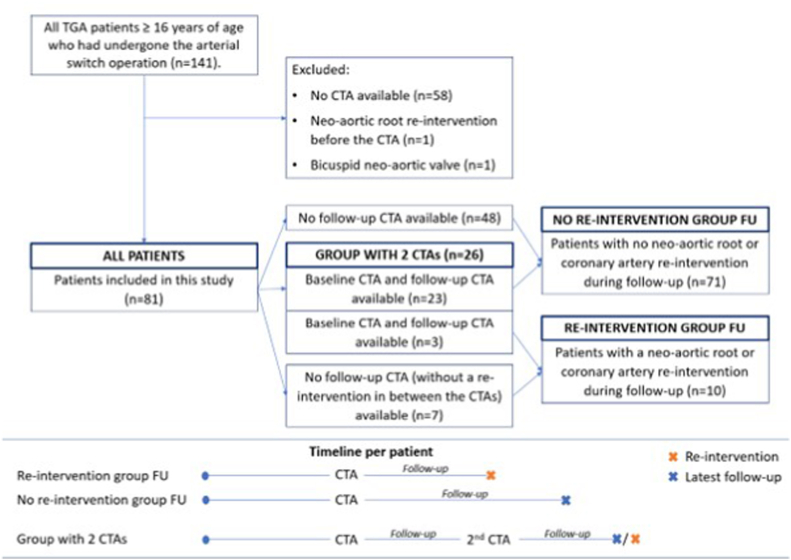
Flow diagram of study design. Abbreviations: CTA, computed tomography angiography; TGA, transposition of the great arteries.

Eighty-three consecutive TGA-ASO patients (≥16 years) were identified. One patient with a bicuspid neo-aortic valve and one patient with neo-aortic root intervention prior to the first CTA were excluded. Eighty-one patients were included for analysis, Fig. 2.

Fifty-six (69 %) patients were male and median age at first CTA was 21.0 [18.5–22.8] years. TGA with an intact ventricular septum (TGA-IVS) was present in 63 (78 %) patients, TGA with a ventricular septum defect (TGA-VSD) in 14 (17 %) patients, and TGA with a double outlet right ventricle (DORV) of the Taussig-Bing type (DORV-TBA), in 3 (4 %) patients. Median age at ASO was 6 [4–22] days and in 74 (91 %) patients the Lecompte maneuver was performed. In one (1 %) patient with pre-ASO anatomy 1LCx-2R, ASO was complicated by a peri-operative myocardial infarction for which conservative treatment was pursued (Table 1).

**Table 1:** Patient demographics and clinical characteristics for all patients, patients with and without an intervention affecting the coronary arteries or the ascending aorta and patients with 2 CTAs, without a re-intervention of the neo-aortic root or coronary artery in between these CTAs.

	All patients (n = 81)	No re-intervention (n = 71)	Re-intervention^a^ (n = 10)	Patients with 2 CTAs (n = 26)	p-value (Intervention vs no intervention)
Sex (male), n (%)	56 (67)	48 (68)	8 (80)	19 (73)	0.716
Age (years), median (IQR)	21.0 (18.5–22.8)	21.1 (18.5–22.8)	20.5 (18.5–20.6)	21.3 ± 4.3	0.747
TGA subtype, n (%)					
TGA-IVS	64 (79)	57 (84)	7 (70)	21 (81)	0.739
TGA-VSD	14 (17)	11 (15)	3 (30)	4 (15)	0.491
DORV-TBA	3 (4)	3 (4)	0 (0)	1 (4)	1.000
Coronary anatomy at birth, n (%)					
1LCx-2R	64 (79)	57 (80)	7 (70)	21 (81)	0.739
1L-2CxR	6 (7)	5 (7)	1 (10)	4 (19)	1.000
Other	3 (4)	2 (3)	1 (10)	1 (0)	0.817
Unknown	8 (10)	7 (10)	1 (10)	0 (0)	1.000
Age ASO (days), median (IQR)	6 (4-22)	6 (3-21)	12.5 (6-32)	6 (3-13)	0.144
Lecompte maneuver, n (%)	74 (91)	64 (90)	10 (100)	24 (92)	0.588
Clinical characteristics					
BMI (kg/m^2^), mean ± SD	22.8 ± 3.7	22.8 ± 3.8	22.8 ± 3.3	23.0 ± 3.8	0.306
BSA (Dubois), mean ± SD	1.9 ± 0.17	1.9 ± 0.17	1.9 ± 0.19	1.9 ± 0.2	0.999
Blood pressure (mmHg), mean ± SD					
Systolic	119 ± 11.7	119 ± 11.9	117 ± 14.4	119 ± 13.1	0.267
Diastolic	69 ± 8.3	69 ± 8.5	69 ± 10.4	68 ± 8.8	0.105
Cardiovascular risk factors, n (%)					
Hypertension	1 (1)	0 (0)	1 (10)	1 (4)	0.233
Family history of premature CAD	15 (19)	15 (21)	0 (0)	6 (23)	0.195
Smoking	9 (11)	5 (7)	4 (40)	2 (8)	**0.011**
Cardiac clinical history, n (%)					
Myocardial infarction	1 (1)	0 (0)	1 (10)	1 (4)	0.123
Ventricular tachycardia	3 (4)	1 (1)	2 (20)	2 (8)	**0.039**
RVOT procedure	12 (15)	10 (14)	2 (20)	6 (23)	0.638
Neo-aortic valve replacement	3 (4)	1 (1)	2 (20)	1 (4)	**0.039**
ICD implantation	3 (4)	1 (1)	2 (20)	2 (8)	**0.039**
Cardiovascular medication, n (%)					
B-blocker	2 (2)	0 (0)	2 (20)	1 (4)	**0.014**
ACEI/ARB	5 (6)	2 (3)	3 (30)	2 (3)	**0.012**
Diuretics	1 (1)	1 (1)	0 (0)	1 (4)	1.000
Anticoagulants	3 (4)	1 (1)	2 (20)	1 (4)	**0.039**
Potential cardiac symptoms, n (%)					
Possible cardiac chest pain	1 (1)	1 (1)	0 (0)	0 (0)	1.000
Non-cardiac chest pain	6 (8)	6 (8)	0 (0)	0 (0)	1.000
Palpitations	3 (4)	3 (4)	0 (0)	0 (0)	1.000
NYHA functional class, n (%)					
NYHA Class I	73 (90)	64 (91)	9 (90)	23 (88)	1.000
NYHA Class II	7 (9)	6 (9)	1 (10)	3 (12)	1.000

Abbreviations: ACEI, angiotensin-converting enzyme inhibitors; ARB, angiotensin receptor blockers; ASO, arterial switch operation; BMI, body mass index; BSA, body surface area; DORV-TBA, DORV of the Taussig-Bing type; ICD, implantable cardioverter-defibrillator; IVS, intact ventricular septum; RVOT, right ventricular outflow tract; TGA, transposition of the great arteries; VSD, ventricular septum defect.

a Type of intervention is detailed in Table 5.

**Fig. 3:**
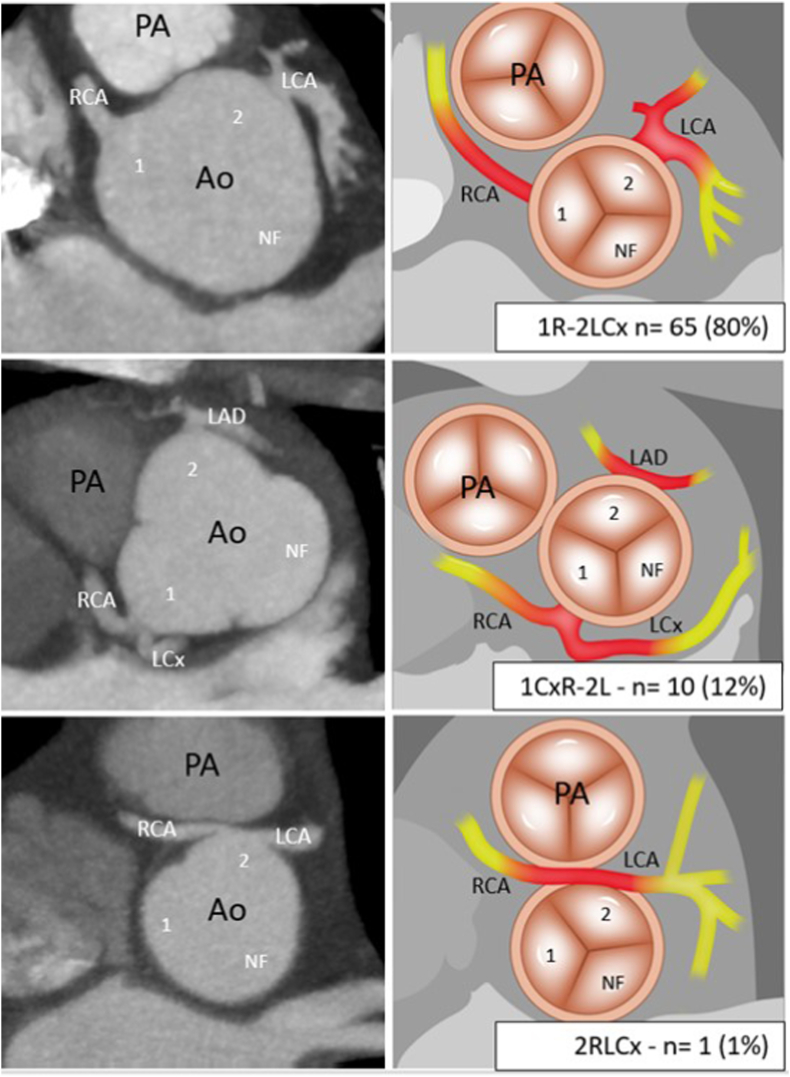
CTA images (left column) and corresponding illustrations (right column) presenting the variants of coronary anatomy in patients with transposition of the great arteries after arterial switch operation classified according to the Leiden Convention coronary coding system Abbreviations: 1, sinus 1; 2, sinus 2; Ao, aorta; CTA, computed tomography angiography; LAD, left anterior descending artery; LCA, left coronary artery; LCx, left circumflex artery; NF, non-facing sinus; PA, pulmonary artery, RCA, right coronary artery.

Indication for baseline CTA was regular follow-up in 68 (84 %) patients, potential cardiac symptoms in 10 (12 %) patients, and other indication in 3 (4 %) patients. Common coronary anatomy (1R-2LCx) postASO was found in 65 (80 %) patients, a retro-aortic Cx originating from the RCA (1CxR-2L) was seen in 10 (12 %) patients and 6 (7 %) patients had another coronary anatomy variant (Fig. 3).

### 4.2 Prevalence of interarterial coronary course, neo-aortic dilatation, and acute angulation at baseline in patients without an intervention on the aorta or coronary arteries

**Fig. 4:**
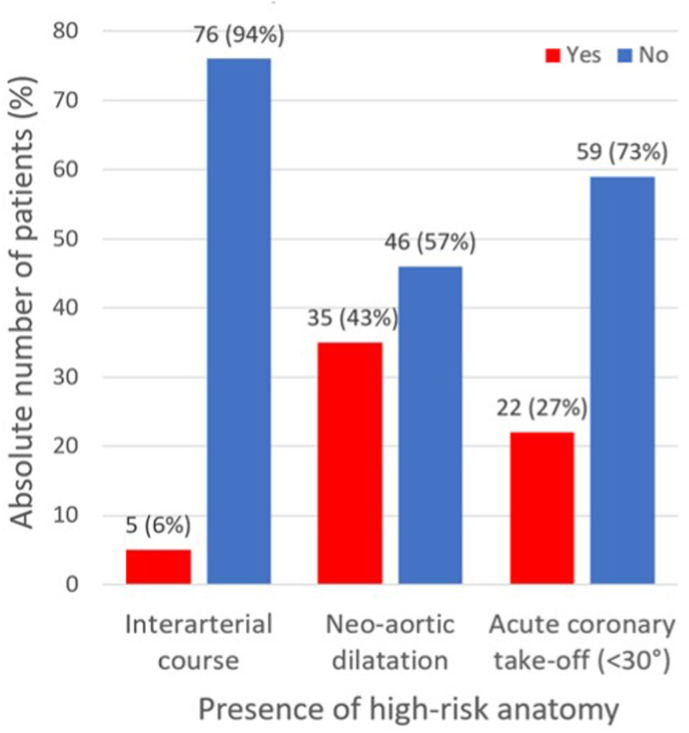
Presence of interarterial course, neo-aortic dilatation (>40 mm) and acute coronary take-off angle (<30°) at baseline CTA Abbreviations: CTA, computed tomography angiography.

**Fig. 5:**
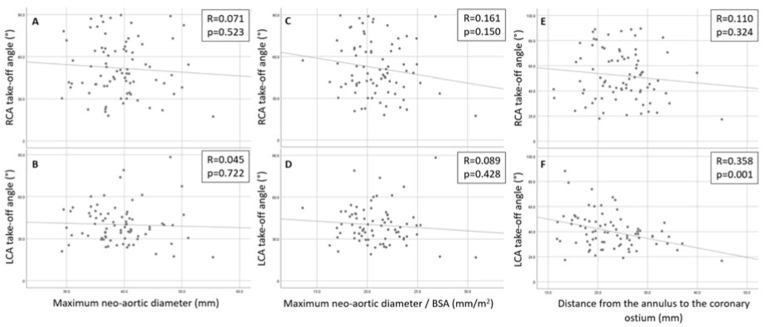
Scatterplots with Pearson’s correlation coefficients illustrating the relation between the coronary take-off angle (°) of the RCA (3A) and LCA (3B) and maximum neo-aortic dimension (mm), the relation between the coronary take-off angle (°) of the RCA (3C) and LCA (3D) and maximum neo-aortic dimension/BSA (mm/m^2^), and the relation between the coronary take-off angle (°) of the RCA (3E) and LCA (3F) and the distance from the annulus to the coronary ostium. Abbreviations: BSA, body surface area; LCA, left coronary artery; RCA, right coronary artery.

In 5 (6 %) patients, an interarterial course of the RCA (n = 2) or LCA (n = 3) was observed, Fig. 4. At baseline, 35/81 (43 %) patients had neo-aortic dilatation (>40 mm). The mean maximum neo-aortic diameter at baseline was 39.2 ± 5.3 mm (range 29.2–55.4 mm) and corrected for BSA 20.9 ± 2.7 mm/m^2^ (range 13.6–30.7 mm/m^2^). Coronary take-off angle of the RCA and LCA were 52.1 ± 19.7° and 40.3 ± 13.8°, respectively. Based on cut-off values of <30° and <45° for an acute coronary take-off angle, 22 (28 %) patients and 61 (76 %) patients met the criteria, respectively (Table 2). Pearson's correlation test revealed no relation between the absolute neo-aortic dimensions and the coronary take-off angle of the RCA (R = 0.07, p = 0.523) and LCA (R = 0.05, p = 0.722) and maximum BSA-corrected neo-aortic dimensions and the coronary take-off angle of the RCA (R = 0.16, p = 0.150) and LCA (R = 0.09, p = 0.428), Fig. 5A–D.

**Table 2:** Baseline CTA measurements for all patients and patients without and with an intervention affecting the coronary arteries or the ascending aorta.

	All patients (n = 81)	No re-intervention (n = 71)	Re-intervention (n = 10)	p-value (Intervention vs no intervention)
Indication CTA, n (%)				
Screening	68 (84)	60 (85)	8 (80)	0.659
Potential cardiac symptoms	10 (12)	10 (14)	0 (0)	0.349
Other	3 (4)	1 (1)	2 (20)	0.039
Coronary anatomy post ASO, n (%)				
1R-2LCx	65 (80)	59 (83)	6 (60)	0.102
1CxR-2L	10 (12)	7 (10)	3 (30)	0.103
Other	6 (7)	5 (7)	1 (10)	0.559
Interarterial course, n (%)				
Yes	5 (6)	4(6)	1 (10)	0.492
Coronary take-off angle (°), mean ± SD				
RCA	52.1 ± 19.7	53.0 ± 13.3	45.9 ± 22.7	0.295
LCA	40.3 ± 13.8	40.3 ± 12.5	40.2 ± 21.7	0.394
Coronary take-off angle, n (%)				
LCA or RCA take-off angle <30°	22 (27)	18 (25)	4 (40)	0.448
LCA or RCA take-off angle <45°	61 (75)	52 (73)	9 (90)	0.437
Maximum neo-aortic diameters (mm), mean ± SD				
Neo-aortic annulus	28.2 ± 3.2	27.9 ± 2.9	30.3 ± 4.2	0.110
Neo-aortic sinus	38.6 ± 5.0	37.6 ± 4.0	45.2 ± 6.3	**0.001**
Level of RCA	37.9 ± 5.6	36.9 ± 4.8	44.5 ± 6.4	**0.001**
Level of LCA	37.9 ± 5.2	37.1 ± 4.4	43.7 ± 6.6	**0.002**
Level of STJ	33.4 ± 5.0	33.2 ± 4.0	35.5 ± 8.3	0.525
Level of ascending aorta	24.5 ± 3.4	24.2 ± 3.1	26.9 ± 4.8	0.146
Max diameter	39.2 ± 5.3	38.2 ± 4.3	46.3 ± 6.5	**0.001**
Maximum neo-aortic diameters/BSA (mm/m^2^), mean ± SD				
Neo-aortic annulus/BSA	15.0 ± 1.6	14.9 ± 1.6	15.7 ± 2.1	0.288
Neo-aortic sinus/BSA	20.5 ± 2.6	20.1 ± 2.2	23.4 ± 3.2	**0.001**
Level of RCA/BSA	20.2 ± 3.0	19.7 ± 2.6	23.1 ± 3.8	**0.005**
Level of LCA/BSA	20.1 ± 2.7	19.8 ± 2.4	22.7 ± 3.7	**0.011**
Level of STJ/BSA	17.8 ± 3.3	17.8 ± 3.2	18.2 ± 3.8	0.916
Level of ascending aorta/BSA	13.1 ± 1.7	13.0 ± 1.6	14.0 ± 2.4	0.266
Max diameter/BSA	20.9 ± 2.7	20.4 ± 2.3	24.0 ± 3.2	**0.001**
Coronary height (mm), median (IQR)				
RCA to neo-aortic annulus	24.9 (20.7–28.6)	24.7 (20.3–28.5)	27.2 (21.3–35.3)	0.217
LCA to neo-aortic annulus	21.5 (18.2–26.3)	21.4 (18.2–26.4)	22.2 (18.1–28.7)	0.869

Abbreviations: BSA, body surface area; CTA, computed tomography angiography; LCA, left coronary artery; Max, maximum; RCA, right coronary artery.

At baseline, median coronary height from the neo-aortic annulus to the RCA ostium was 24.9 mm [20.7–28.6 mm] and to the LCA ostium was 21.5 mm [18.2–26.3 mm]. Although statistically significant, the relation between ostium height and coronary take-off angle of the LCA was poor (R = 0.36, p = 0.001), Fig. 5E. For the RCA this relation was not observed (R = 0.11, p = 0.324), Fig. 5F.

### 4.3 Follow up CTA in patients without an intervention of the aorta or coronary arteries

A subgroup of twenty-six (32 %) patients had a follow-up CTA without a coronary artery or (neo)aortic re-intervention in between the CTAs, Fig. 2. Mean time between the CTAs was 7.9 ± 3.1 years. In this subgroup, average maximum neo-aortic diameter at baseline was 38.9 ± 4.6 mm (range 29.5–48.4 mm), Table 3. The average maximum neo-aortic diameter at follow-up CTA was 40.0 ± 5.0 mm (range 30.7–51.7) which was significantly larger (p = 0.001). Increase of neo-aortic dimensions was measured at the levels of the neo-aortic annulus, neo-aortic sinus, RCA ostium and LCA ostium, sinotubular junction, and ascending aorta with average annual growth rates of 0.06 ± 0.12 mm, 0.16 ± 0.20 mm, 0.16 ± 0.22 mm, and 0.12 ± 0.1 6 mm, 0.07 ± 0.14 mm, and 0.10 ± 0.19 mm, respectively. Mean coronary take-off angles at baseline of the RCA and LCA were 53.1 ± 19.3° and 40.9 ± 14.5°, respectively. Follow-up CTA showed a RCA take-off angle of 54.4 ± 19.3° and a LCA take-off angle of 40.9 ± 14.0°. The coronary take-off angle between the baseline CTA and follow-up CTA of the RCA (p = 0.155) and LCA (p = 0.692) was not significantly different.

**Table 3:** Computed tomography angiography indication and measurements of all patients with 2 CTAs and without a re-intervention of the neo-aortic root or coronary artery in between the CTAs.

	Baseline CTA	Follow-up CTA	p-value
Indication CTA, n (%)			
Screening	26 (100)	23 (89)	0.257
Cardiac complaints	0 (0)	2 (8)	0.157
Other	0 (0)	1 (4)	0.655
Coronary anatomy post ASO, n (%)			
1R-2LCx	21 (81)	21 (81)	1.000
1CxR-2L	4 (15)	4 (15)	1.000
Other	1 (4)	1 (4)	1.000
Interarterial course, n (%)			
Yes	2 (8)	2 (8)	1.000
Coronary take-off angle (°), mean ± SD			
RCA	53.1 ± 19.3	54.4 ± 19.3	0.155
LCA	40.9 ± 14.5	40.9 ± 14.0	0.692
Coronary take-off angle, n (%)			
LCA or RCA take-off angle <30°	7 (27)	7 (29)	0.564
LCA or RCA take-off angle <45°	20 (77)	20 (80)	0.157
Neo-aortic diameters (mm), mean ± SD			
Neo-aortic annulus	28.4 ± 2.9	28.8 ± 3.0	**0.036**
Neo-aortic sinus	38.6 ± 4.4	39.8 ± 5.0	**0.000**
Level of RCA	37.1 ± 4.4	38.4 ± 4.9	**0.001**
Level of LCA	37.6 ± 4.7	38.4 ± 5.0	**0.002**
Level of STJ	31.3 ± 5.5	32.3 ± 5.5	**0.007**
Level of ascending aorta	23.6 ± 3.6	25.1 ± 3.6	**0.003**
Max diameter	38.9 ± 4.6	40.0 ± 5.0	**0.001**
Neo-aortic diameters (mm/m^2^), mean ± SD			
Neo-aortic annulus/BSA	15.0 ± 1.3	14.5 ± 1.3	**0.003**
Neo-aortic sinus/BSA	20.5 ± 1.7	20.2 ± 2.0	0.144
Level of RCA/BSA	19.8 ± 2.1	19.5 ± 2.2	0.238
Level of LCA/BSA	20.0 ± 2.2	19.6 ± 2.3	**0.045**
Level of STJ/BSA	16.8 ± 2.3	16.4 ± 2.3	0.092
Level of ascending aorta/BSA	12.7 ± 1.4	12.6 ± 1.5	0.647
Max diameter/BSA	20.7 ± 1.8	20.3 ± 2.0	0.082
Coronary height (mm), median (IQR)			
RCA to neo-aortic annulus	24.8 (20.1–27.6)	24.3 (19.5–28.4)	**0.026**
LCA to neo-aortic annulus	23.1 (18.6–26.0)	23.4 (18.3–26.1)	0.626

Abbreviations: BSA, body surface area; CTA, computed tomography angiography; LCA, left coronary artery; Max, maximum; RCA, right coronary artery.

### 4.4 Comparison of baseline characteristics and CTA measurements of patients with and without a re-intervention during follow-up

Ten (12 %) patients had a re-intervention for coronary artery or (neo)aorta during the follow-up period after baseline CTA and were stratified into the ‘intervention’ group. Seventy-one (88 %) patients had no coronary artery or (neo)aortic re-intervention after baseline CTA and were stratified into the ‘no intervention’ group, Table 4. Re-intervention indications consisted of neo-aortic root surgery in 8 (10 %) patients and CABG or PCI due to coronary obstruction in 2 (2 %) patients. At baseline, patient age, gender, TGA subtype and coronary anatomy at birth did not differ significantly between patients without and with an intervention affecting the coronary arteries or the ascending aorta. The number of patients with a history of neo-aortic valve replacement (p = 0.039), ventricular tachycardia (p = 0.039) and implantable cardioverter-defibrillator (ICD) implantation (p = 0.039) before baseline CTA was significantly higher in patients with an intervention, Table 1. The characteristics of patients with a re-intervention are individually presented in Table 5. Patient 1 and 2 had a coronary artery re-interventions and patient 3–10 had a neo-aortic re-intervention.

**Table 4:** Clinical events and re-interventions during follow-up for all patients and patients without and with an intervention affecting the coronary arteries or the ascending aorta.

	All patients (n = 81)	No re-intervention (n = 71)	Re-intervention (n = 10)	p-value (Re-intervention vs no re-intervention)
Follow-up duration (years), median (IQR)	6.5 (3.5–10.3)	5.7 (3.3–10.1)	10.1 (8.1–11.9)	**0.009**
Clinical events, n (%)				
Out-of-hospital cardiac arrest	1 (1)	0 (0)	1 (10)	0.123
Endocarditis	1 (1)	1 (1)	0 (0)	0.877
VT/VF	2 (3)	1 (1)	1 (10)	0.233
Re-interventions, n (%)				
RVOT procedure	3 (4)	2 (3)	1 (10)	0.330
Neo-aortic root intervention	8 (10)	0 (0)	8 (80)	**0.000**
Neo-aortic valve replacement	7 (8)	0 (0)	7 (70)	**0.000**
Coronary intervention	2 (3)	0 (0)	2 (20)	**0.014**
Mitral valve repair	1 (1)	0 (0)	1 (10)	0.123
VT ablation	1 (1)	0 (0)	1 (10)	0.123
ICD implantation	1 (1)	0 (0)	1 (10)	0.123
PM implantation	1 (1)	0 (0)	1 (10)	0.123

Abbreviations: ICD, implantable cardioverter-defibrillator; PM, pacemaker; RVOT, right ventricular outflow tract; VF, ventricular fibrillation; VT, ventricular tachycardia.

**Table 5:** Individual baseline characteristics, operative report information, CTA measurements and follow-up data of the 10 patients that underwent a re-intervention.

Study patient	1	2	3	4	5	6	7	8	9	10
TGA/ASO demographics										
***TGA subtype***	TGA-IVS	TGA-IVS	TGA-IVS	TGA-IVS	TGA-VSD	TGA-VSD	TGA-IVS	TGA-IVS	TGA-IVS	TGA-VSD
***Coronary anatomy at birth***	1L-2CxR	1L-2CxR	1LCx-2R	1LCx-2R	1L-2CxR	1LCx-2R	1RL-2Cx	1LCx-2R	1L-2CxR	1LCx-2R
***Age at ASO (days)***	15	12	7	2	5	100	9	5	13	111
***Lecompte maneuver***	Yes	Yes	Yes	Yes	Yes	Yes	Yes	Yes	Yes	Yes
***Operative report***										
***Coronary transfer technique***	Unclear	Button technique	Button technique	Unclear	Button technique	Button technique	RCA/LAD button technique, Cx trap-door technique	LCA trap-door technique, RCA button technique	LCA trap-door technique, RCA button technique	Button technique
***Specific surgical remarks***	Perioperative LCx hypoperfusion	NR	NR	NR	Dilated AP (neo-aorta)	Dilated AP (neo-aorta)	NR	NR	Dilated AP (neo-aorta)	Dilated AP (neo-aorta)
**Patient characteristics**										
***Age at baseline (years)***	18	19	23	18	19	28	17	20	19	18
***Gender***	Female	Male	Male	Male	Male	Male	Male	Male	Male	Female
***Relevant cardiac history***	Perioperative myocardial infarction in LCx territory, ventricular tachycardia and ICD implantation.	None	Severe neo-aortic regurgitation, VT and ICD implantation.	Mechanical AVR	Balloon valvuloplasty of LPA stenosis.	Mechanical AVR	None	None	None	None
***Blood pressure (mmHg)***	120/60	110/65	130/80	110/80	105/60	100/60	119/77	100/60	140/85	135/60
***BMI (kg/m***^***2***^***)***	18.8	20.4	24.1	21.2	21.8	27.5	18.5	22.3	26.3	27.2
***BSA (m***^***2***^***)***	1.73	1.68	1.85	1.79	1.94	2.16	1.8	2.01	2.23	2.12
**CTA characteristics**										
***Indication***	Screening	Screening	Other: Evaluation of coronary stenosis due to history of ventricular tachycardia and mild LV dysfunction with hypokinesia mid-anteroseptal.	Screening	Screening	Screening	Other: Evaluation of aortic dimensions.	Screening	Screening	Screening
***Coronary anatomy (Leiden Convention)***	1R-2LCx	1CxR-2L	1R-2LCx	1R-2LCx	1CxR-2L	1R-2LCx	1Cx-2RL	1R-2LCx	1CxR-2L	1R-2LCx
***Interarterial course***	Yes	No	No	No	No	No	No	No	No	No
***RCA take-off angle (°)***	22.4	24.2	41.1	88.9	73.0	54.3	17.4	52.7	46.8	38.2
***LCA take-off angle (°)***	32.2	24.1	39.8	88.3	39.6	30.6	16.8	36.7	26.4	67.4
***Max aortic diameter (mm)***	33.7	36.8	46.9	48.0	48.4	51.1	55.4	44.4	48.2	50.0
***Max aortic diameter/BSA (mm/m***^***2***^***)***	19.4	21.9	25.3	26.8	24.9	23.6	30.8	22.1	21.6	23.6
***RCA height to annulus (mm)***	21.1	15.4	24.8	26.7	27.6	39.9	44.9	28.5	21.3	33.7
***LCA height to annulus (mm)***	17.9	18.2	19.0	13.4	21.4	37.0	44.9	25.9	23.3	23.0
**Follow-up**										
***Age at intervention (years)***	27	19	24	26	28	32	18	21	30	18
***Indication intervention***	Multiple VT episodes, CAG: external compression ostium RCA + mitral regurgitation.	CT: Acute coronary take-off angle with 50–60 % stenosis in proximal LCA.	Severe aortic regurgitation and aortic dilatation (max 48 mm).	Progressive neo-aortic dilatation (max 54 mm) + mild pressure gradient across the AVR (30 mmHg)	Neo-aortic root dilatation (max 55 mm)	Progressive aortic root dilatation (max 51 mm) + severe mitral regurgitation	Severe aortic dilatation (max 55 mm) + moderate aortic regurgitation	Severe aortic regurgitation + aortic dilatation (max 44 mm) + LV dilatation	Severe aortic regurgitation + aortic root dilatation (max 48 mm) + severe LV dysfunction	Aortic root dilatation (max 50 mm) + moderate-severe aortic regurgitation
**Intervention**	CABG (RIMA-RCA) + MVP	PCI LCA	Bentall procedure	Bentall procedure + re-AVR	Pears procedure	Bentall procedure + MVP	Bentall procedure	Bentall procedure + AVR	Bentall procedure + AVR + patchplasty MPA and LPA	Bentall procedure + AVR

Abbreviations: ASO, arterial switch operation; AVR, aortic valve replacement; BMI, body mass index; BSA, body surface area; CABG, coronary artery bypass graft; CAG, coronary angiography; CTA, computed tomography angiography; ICD, implantable cardioverter-defibrillator; IVS, intact ventricular septum; LAD, left anterior descending artery; LCA, left coronary artery; Cx, circumflex artery; LPA, left pulmonary artery; LV, left ventricular; MPA, main pulmonary artery; MVP, mitral valve plasty; NR, none reported; PA, pulmonary artery; PCI, percutaneous coronary intervention; RCA, right coronary artery; RIMA, right internal mammary artery; TGA, transposition of the great arteries; VSD, ventricular septum defect; VT, ventricular tachycardia.

The prevalence of an interarterial course was not significantly different between patient with and without a re-intervention during follow-up after baseline CTA (p = 0.492). CTA measurements did not show a significant difference in coronary take-off angle between patients with and without an intervention for the RCA (p = 0.295) and LCA (p = 0.394) (Table 2). However, mean neo-aortic diameters at baseline were significantly larger in patients with an intervention compared to patients without an intervention (p ≤ 0.002). Furthermore, all patients with a re-intervention compared to 36 (53.7 %) of the patients without a re-intervention met the criteria for neo-aortic dilatation (neo-aortic diameter of >40 mm) and/or an acute coronary take-off angle (<30°) (Table 2).

*Coronary interventions.* Two patients underwent coronary artery interventions during follow-up. A CABG (RIMA to proximal RCA) was performed one patient (27 years) who experienced several episodes of fast ventricular tachyarrhythmias indicative for an ischemic trigger from the RCA effluence territory. Coronary CTA showed an interarterial RCA (1R-2LCx), with RCA angle take-off of 22.4° and a normal neo-aortic root of diameter of 33.7 mm (19.4 mm/m^2^). In a second patient, who was asymptomatic, PCI of the ostial segment of the LCA was performed (1CxR-2L) after surveillance coronary CTA revealed a take-off angle of 24.1° with significant narrowing of the LCA ostium. Maximum neo-aortic diameter in this patient was 36.8 mm (21.9 mm/m^2^).

*Neo-aortic interventions.* Eight patients underwent neo-aortic interventions during follow-up. All of these patients had neo-aortic dilatation (>40 mm) at baseline CTA. Characteristics of these patients and the intervention are presented in Table 5.

### 4.5 Interobserver variability

Interobserver variability analysis for measurements of the coronary take-off angle and neo-aortic dimensions showed good reproducibility for both measurements. For the coronary take-off angle the ICC was 0.865 (p < 0.001) for individual measurements and for the neo-aortic dimensions the ICC was 0.900 (p < 0.001) for individual measurements.

## 5 Discussion

This retrospective study of coronary anatomy during adulthood in TGA patients late after ASO revealed a prevalence of 43 % of neo-aortic dilatation, a prevalence of 6 % of an interarterial coronary course and a prevalence of 27 % of an acute take-off angle (<30°) of at least one coronary artery at baseline at a median duration of 21.0 years post ASO. All patients who had a (neo)aortic re-intervention met the criteria for neo-aortic dilatation (neo-aortic diameter of >40 mm) and all patients who had a coronary artery re-intervention had an acute coronary take-off angle (<30°) at baseline CTA.

### 5.1 Neo-aortic dilatation, interarterial coronary course and acute coronary take-off angle in adult TGA-ASO patients

In ASO patients, larger neo-aortic dimensions have previously been related to diminished amount of collagen in the neo-aortic root [20]. In this cohort, consisting of 81 patients, neo-aortic dimensions with a mean neo-aortic diameter of 38.6 mm and corrected for BSA of 20.5 mm/m^2^ at baseline were observed. These neo-aortic diameters concur well with other long-term follow-up studies in ASO patients [21,22], but are larger compared to healthy controls, in which the absolute mean aortic root diameter for men is 31.8 ± 3.7 (indexed 16.5 ± 2.2) and for woman 28.5 ± 3.0 (indexed 17.1 ± 2.1) at a mean age of 44.7 years [23,24].

Moreover, the current study demonstrates a high prevalence of high-risk anatomical features (acute coronary take-off angle and interarterial course) in TGA-ASO patients. These results correspond with findings from Michalak et al. who reported a prevalence of 8 % for an interarterial course and a prevalence of 50 % for ASO patients with an acute coronary take-off angle (<30°) [6]. Furthermore, concurring with our results, lower coronary take-off angles were observed in the LCA compared to the RCA. An explanation for the high prevalence of patients with an acute coronary take-off angle may be the re-implantation technique used for the coronary arteries during ASO. To avoid coronary kinking during the ASO, the height at which the coronary arteries are re-implanted into the neo-aorta can be adjusted, which could in turn affect coronary take-off angle. This is partly supported by data in our study, that showed that mean coronary ostium height was higher compared to the general population [25]. We found a significant, yet weak correlation between the coronary ostium height of the LCA and coronary take-off angle. This correlation was not observed for the ostium height of the RCA and coronary take-off angle.

Our data could not confirm the previous suggestion that the neo-aortic root diameter may be associated with a more acute take-off angle [6,7]. Potential reason for the discrepancy with our previous report may be that a substantially larger group of patients was analyzed in the current study, and a more robust method is currently used to measure the coronary take-off angle [17,18]. Moreover, neo-aortic dimensions were only mildly increased in our cohort at a median age of 21.0 years post ASO. Although we cannot exclude that more critical aorta dilatation can contribute to an increase in coronary angulation, there is currently no data to support this, at least in non-critical neo-aortic dimensions [6].

### 5.2 Neo-aortic dimensions and the coronary take-off angle during follow-up in patients without re-intervention between the CTAs

To the best of our knowledge, this is the first serial CTA follow-up study in adult patients with a diagnostic interval of nearly 8 years evaluating change of neo-aortic diameter and coronary angulation over time. We reported a small but significant increase of maximum neo-aortic dimensions with an annual mean growth rate of the neo-aortic root dimensions of 0.16 mm over this 8-year follow-up period in a subgroup of 26 patients. This neo-aortic growth rate is almost a factor 2 higher compared to the estimated annual aorta growth rate of 0.08 mm in healthy adults [26]. The annual aortic growth rate in the current study was lower compared to the study by van der Palen et al. [9]. A possible explanation could be that the annual growth rate determined in that study was estimated from longitudinal data from birth to adulthood. This might have led to an overestimated neo-aortic growth rate in adulthood in the study of van der Palen et al. [9]. In addition, as annual growth cannot reliably be measured after a neo-aortic intervention, e.g. Bentall procedure, we only included patients without neo-aortic or coronary re-interventions between the baseline and latest CTA. This could result in an underestimation of the neo-aortic growth rate in the overall cohort.

### 5.3 Patients with a neo-aortic or coronary artery re-intervention

Re-intervention of the neo-aortic root occurred in 8 (10 %) patients and 2 (2 %) patients underwent coronary re-intervention, during median follow-up of 6.5 years. These re-intervention rates are consistent with a recent long-term follow-up study [27]. In our cohort, coronary re-intervention was performed because of partial coronary artery obstruction. Coronary CTA in both patients showed an acute coronary artery angulation and in one patient the RCA had an interarterial course. Although the number of coronary complications was low in our cohort, presence of coronary high-risk features in both these patients emphasizes the importance of identification of patients with these features. In our cohort, all patients with a neo-aortic root re-intervention during follow-up had a maximum neo-aortic diameter of >40 mm and all patients with a coronary re-intervention a coronary take-off angle of <30 on baseline CTA.

### 5.4 Coronary high-risk anatomical features

Currently, primary evaluation of the coronary artery anatomy and patency is performed by CTA and no consensus in international guidelines is reached concerning optimal follow-up strategy and frequency for evaluation of re-implanted anomalous coronary arteries late after ASO [5,28]. We recently performed a systematic literature review and reported coronary complications in 0.8 % of TGA patients after ASO during adulthood [8]. This is slightly lower than the coronary complication rate of 2.5 % in the current study. Exact pathophysiology causing coronary complications is ambiguous. Literature shows that presence of coronary high-risk anatomical features, including acute take-off angle, has been associated with myocardial ischemia and sudden death in athletes [29,30]. After ASO, partial or total coronary obstruction may present less symptomatic due to denervation caused by damage to the cardiac plexus during transection of the great arteries, which may halt early recognition [31,32]. In our cohort, this phenomenon potentially occurred in one asymptomatic patient who had an acute take-off angle and proximal narrowing of the LCA ostium with ischemia on the SPECT for which a PCI was performed. Therefore, understanding the pathophysiology of coronary complications late after ASO and early recognition of coronary complications through diagnostic tools is crucial.

### 5.5 Limitations

This is a retrospective cohort study and is therefore subject to intrinsic limitations related to the nature of the design. The number of patients are reflective of the relative rarity of this condition. The results of these analyses should be interpreted in the context of larger cohorts including patients from multiple surgical teams with a wider range of neo-aortic dimensions which may be observed during a longer follow-up period. Nevertheless, this is the largest cohort of TGA-ASO patients with serial CTA follow-up for coronary anatomy and neo-aortic diameters during adulthood.

## 6 Conclusion

This study reports a prevalence of 43 % of neo-aortic dilatation, 6 % of interarterial coronary course and 27 % for acute coronary take-off angle in adult patients at 21.0 years post ASO.

All patients who had a (neo)aortic re-intervention met the criteria for neo-aortic dilatation (neo-aortic diameter of >40 mm) and all patients who had a coronary artery re-intervention had an acute coronary take-off angle (<30°) at baseline CTA. This hypothesis generating study suggests that an active surveillance in patients with neo-aortic dilation and/or an acute angulation of < 30° post ASO might be considered and requires prospective evaluation.

## Funding statement

We acknowledge the support from the Netherlands Cardiovascular Research lnitiative: An initiative with support of the 10.13039/100002129Dutch Heart Foundation and Hartekind, CVON2019-002 OUTREACH.

Institutional Review Board (IRB) approval.

## Informed consent statement

The need for written informed consent was waived by the institutional medical ethical board, The Medical Ethics Committee Leiden, The Hague, Delft.

## Data availability statement

Data are available upon reasonable request.

## Declaration of competing interest

The authors declare that they have no known competing financial interests or personal relationships that could have appeared to influence the work reported in this paper.
